# Self-reported current sleep behaviors of adult athletes from different competitive levels and sports

**DOI:** 10.5935/1984-0063.20200044

**Published:** 2021

**Authors:** Rebecca K Randell, Rosie Anderson, James M Carter, Ian Rollo

**Affiliations:** 1 Life Sciences, Gatorade Sports Science Institute - PepsiCo Inc. - Leicester - United Kingdom.; 2 Loughborough University, School of Sport, Exercise and Health Sciences -Loughborough - Leicestershire - United Kingdom.

**Keywords:** Sleep Behaviors, Sleep Quality, Team Sports, Individual Sports, Adult Athletes

## Abstract

**Objectives:**

To quantify self-reported current sleep behaviors in a range of adult athletes. In addition, to determine any differences in sleep duration and sleep quality, depending on sport type and competitive level.

**Material and Methods:**

In this cross-sectional study, 313 athletes (243 male, 70 female), competing in a variety of sports and competitive level, completed the Pittsburgh sleep quality index (PSQI) and a questionnaire which captured current sleep behaviors. Sleep quality was calculated using the global PSQI score (≥ 5 indicative of poor sleep quality).

**Results:**

On average, athletes self-reported sleep duration was 7:34 ± 1:00 h:min. Overall, 19% of athletes achieved less than 7 h of sleep, 50% achieved less than 8 h. Global PSQI score was 5.0 ± 2.4, with poor sleep quality found in 55% of athletes. Sleep duration was significantly shorter in runners compared to basketball, soccer and rugby players (p < 0.05). Recreational athletes slept significantly less (7:08 ± 0:54 h:min) than competitive (7:32 ± 1:00 h:min), national (7:50 ± 1:00 h:min) and elite level athletes (7:49 ± 0:51 h:min). No differences in sleep quality were found between sport or competitive level.

**Discussion:**

Half of the athletes failed to achieve 8 h of sleep per night and the majority reported compromised sleep quality. Sport type and competitive level may influence sleep duration; however, these factors do not seem to cause discrepancies in sleep quality. This study provides novel data into the sleep behaviors of adult athletes, and suggests strategies to improve sleep duration and quality may be warranted.

## INTRODUCTION

Sleep aids recovery following demanding physical and mental activity, and is considered a fundamental process for athletes^1,2^. Healthy adults are advised to acquire 7-9 h of sleep per night^3^, with 8 h of sleep per night shown to prevent neuro-behavioral deficits linked to sleep restriction^4^. Alongside sleep duration, sleep quality is an important consideration and refers to the number of sleep disturbances experienced during sleep^5^. Although regular exercise is associated with better sleep quantity and quality^6^, paradoxically, athletes have been reported to experience worse sleep quality (longer sleep latencies, greater sleep fragmentation, non-restorative sleep), in comparison to the general population^7^.

Impaired sleep has important implications for athletes’ physical and technical performance^8^. Restricting sleep by 33% (5 h) was associated with a deterioration of skill, tennis serve, which could not be rescued by caffeine ingestion prior to exercise^9^. More extreme sleep deprivation (30 h) has been linked to lower muscle glycogen concentrations in team sport athletes, resulting in slower self-selected running speeds and reduced sprint times during 50 min of intermittent running^10^. Furthermore, a recent study reported an association between sleep quality and injury occurrence^11^. In this study sleep variables, measured using sleep diaries and wrist activity monitors, were monitored in 23 elite male soccer players over a 6 month period. These authors found a moderate negative correlation between sleep efficiency and several injury parameters such as absence time, injury severity, and number of injuries^11^. Additionally, a study conducted in adolescent athletes reported that athletes who slept for <8 h per night were 1.7 times more likely to experience an injury, compared to those individuals who slept for >8 h^12^. Although these data are causational, the link between sleep duration/quality and injury should not be dismissed.

In addition, sleep deprivation is associated with negatively influencing athlete’s psychological. Blumert et al.^13^ observed negative mood disturbances following acute (24 h) sleep loss in male weightlifters, when compared to a normal night sleep. Furthermore, when sleep is restricted to 50% of habitual time, over a longer period (12 days), athletes experience a 3% increase in generalized body pain, back pain, and stomach pain^14^.

Conversely, extended sleep duration is linked with improvements in athletic physical performance and athlete’s mood^15^. As such, there has been interest in assessing sleep quantity and quality of athletes. When compared to non-athlete controls, Bender et al.^24^ found National level athletes had reported poorer sleep quality, assessed using the Pittsburgh sleep quality index (PSQI). When looking at sleep variables between sport types, Lastella et al.^16^ recorded sleep data using self-report sleep diaries and wrist activity monitors from 124 athletes competing in five individual sports and four team sports. These authors reported significantly shorter sleep durations and poorer sleep efficiency in individual sport athletes compared to team sport athletes^16^. Indeed, athletes from specific sports may be at greater risk of sleep disturbance due to the differing training, travel, and competition demands^7,16^. A recent study by Anderson and Reale^17^ found no main effect of sport on amount of sleep per night, and did find a correlation between self-reported training time and sleep duration. However, this data was collected from adolescent athletes, and largely consisted of team sport athletes.

To date, no studies have assessed the difference in sleep behaviors between adult athletes competing at different competitive levels (i.e., elite, national or recreational level). Understanding sleep behaviors of athletes from different competitive levels may help inform bespoke interventions or education, that directly target sleep quantity and quality for athletes. In addition, there are few studies assessing the association between age, sex and sport type on sleep quality and duration. Therefore, the aims of this study are threefold. Firstly, to quantify self-reported current sleep duration and quality in a range of adult athletes competing at different levels and in a wide range of sports. Secondly, to compare self-reported sleep behaviors with self-reported ideal sleep behaviors; and, finally, to determine if differences exist between sleep duration/quality and sex, age, sport type, and competitive level. It was hypothesized that sleep quality will be lower in athletes who compete at higher competitive levels, and also in athletes who compete in an individual sport. Furthermore, it was hypothesized that athletes will achieve sleep less than the recommended guidelines, and that the athletes current sleep duration will be less than their ideal.

## MATERIAL AND METHODS

### Participants

The participant population consisted of 313 male (N=243) and female (N=70) athletes from a variety of sports (Table 1) (mean age ± SD; 27 ± 8 y, age range 18 - 54 y). All participants were recruited via e-mail, personal visits/meetings, telephone calls, or the athlete personally contacting the testing facility. The competitive level of the participants was self-reported and categorized into recreational, competitive, national or elite. Athlete characteristics, grouped by competitive level, can be found in Table 2.

**Table 1:** Sports frequencies by sex.

Sport	N	Male	Female	Sport	N	Male	Female
Triathlon	40	19	21	Cricket	4	4	0
Running	39	26	13	Motor Sports	3	3	0
Rugby Union	38	38	0	Squash	3	3	0
Cycling	37	36	1	American football	3	3	0
Soccer	23	22	1	Tennis	2	2	0
Basketball	21	10	11	Golf	2	2	0
Track and Field	18	6	12	Gymnastics	0	0	1
Field Hockey	18	17	1	Handball	1	1	0
Rugby League	15	15	0	Rowing	1	1	0
Fitness	13	11	2	Skiing	1	1	0
Paddle Sports	8	8	0	Swimming	1	0	1
Martial Arts	7	7	0	Duathlon	1	1	0
Occupational	7	6	1	Weightlifting	1	1	0
Boxing	4	0	4	Netball	1	0	1

Sports in the grey area represent those that had a frequency above 20 and were included in the comparative analysis between sports.

**Table 2:** Self-reported training information, and current and ideal sleep behaviors.

N	Elite	National	Competitive	Recreational
	37	76	149	51
Males/Females	26 / 11	62 / 14	119 / 30	36 / 15
Age (y)	27 ± 7b	22 ± 5c	27 ± 8b	33 ± 9a
Sleep onset latency (min)	22 ± 13	23 ± 20	21 ± 15	20 ± 20
# training sessions/per week	7 ± 2a	6 ± 2ab	5 ± 2b	4 ± 2c
Hours of training per week (min)	127 ± 77a	97 ± 35b	92 ± 36b	67 ± 26c
≤15 min	32.4%	52.6%	49.0%	58.8%
16-30 min	59.5%	34.2%	36.2%	33.3%
31-60 min	8.1%	7.9%	14.8%	5.9%
>60 min	0.0%	5.3%	0.0%	2.0%
Current Sleep Duration (h:min)	07:49 ± 00:50a	07:49 ± 01:02a	07:32 ± 01:00ab	07:07 ± 0:56b
Current bedtime (h:min)	22:31 ± 00:46b	23:13 ± 00:55a	23:02 ± 00:54a	22:40 ± 00:39b
Current wake time (h:min)	07:11 ± 00:46bc	07:49 ± 01:05a	07:33 ± 01:17ab	06:37 ± 00:52c
Ideal sleep duration (h:min)	09:23 ± 00:53ab	09:43 ± 01:05a	09:26 ± 00:55ab	09:03 ± 00:51b
Global PSQI score	4.9 ± 1.9a	5.0 ± 2.3a	5.2 ± 2.4a	4.9 ± 3.0a

Values are mean±SD. Groups sharing the same letter are not significantly different (p<0.05).

Study inclusion criterion included: age 18-60 y, participation in ≥1 session per week of sporting activity, healthy (assessed using a general health questionnaire), and no known cardiovascular or metabolic disorders. On average, the athletes took part in 5 ± 2 training session per week, lasting 93 ± 44 min. The study was approved by the Loughborough University Ethics Approvals (Human Participants) Sub-Committee and all participants provided written informed consent.

### Questionnaires

This cross-sectional study involved participants completing two sleep-related questionnaires: the Pittsburgh sleep quality index (PSQI)^18^ and a questionnaire which captured usual sleep behaviors. Data was collected during a single visit to the Gatorade Sports Science Institute (GSSI) laboratory at Loughborough University, UK, over a two-year period from 2012 to 2014. Due to the nature of the study, we were unable to collect objective measures of sleep behavior; therefore, we opted to use questionnaires as they are commonly used to provide a general measure about the subjective duration and quality of sleep^19^. Furthermore, the PSQI is a validated questionnaire used to establish self-reported current sleep behaviors including bedtime, sleep onset latency, wake time, sleep duration, and sleep quality (questions 1-4)^18^. The questionnaire generates a score (using a 0-3 scale) for 7 different components; sleep quality, sleep latency, sleep duration, habitual sleep efficiency, sleep disturbances, use of sleep medication, and daytime dysfunction. Overall sleep quality is measured using the global PSQI score, which represents a combination of the7 component scores listed (a score of ≥5 is indicative of poor sleep quality). The second questionnaire was used to determine self-reported ideal bedtime (“To feel my best, I should go to bed at_ ”), wake time (“To feel my best, I should get up at_”), and calculated sleep duration. In addition, this questionnaire captured self-reported daily training time and duration (“I usually exercise at (insert time) for (insert time) minutes”). If any answer was written as a range (e.g., 07:00-08:00 a.m., or 90-120 min), the midpoint was used for the analysis.

### Statistical analysis

Current and ideal sleep duration, wake time and bedtime, as well as sleep quality, were the dependent variables. The independent variables were sport type, competitive level, age, sex, number of training days, and training duration. Data analysis was completed using Minitab^®^ 17 Statistical Software. All data was checked for normality and were considered normally distributed if *p*>0.05 on the Shapiro-Wilk test. A paired t-test was performed to compare current and ideal sleep duration. A 2-sample t-test was used to identify sex differences in current sleep behaviors (sleep onset latency, bedtime, wake time, and duration) and quality. In addition, a one-way ANOVA test was performed to determine differences in sleep duration and quality between sports (only sports with N ≥ 20 were included in analysis) (Table 1). In addition, a one-way ANOVA was used to compare differences in sleep behaviors and quality between athletes’ competitive level and age. The categories for competitive level were; Elite (N=37), National (N=76), Competitive (N=149) and Recreational (N=51). Age groups were categorized as 18-24, 25-34, 35-44, and 45-55 years old. A sleep duration of ≥7 hours was used as a cutoff for optimal sleep^3^. Pearson correlation coefficient (r) as well as coefficient of determination (r^2^) values were used to assess the relationship between age and: current bedtime, wake time, sleep duration and global PSQI score. In addition, a Pearson correlation coefficient (r) and coefficient of determination (r^2^) values were also used to assess potential associations between daily training duration (min) and sleep duration and sleep latency, as well as the number of training sessions per week and sleep duration and sleep latency.

## RESULTS

The PSQI questionnaire revealed that for all athletes (N=313) current self-reported bedtime (hh:mm) was 22:57 ± 00:53 (range 20:30-02:00) and self-reported wake time (hh: min) was 07:25 ± 01:11 (range 04:00 - 12:00), resulting in an average sleep duration of 7:34 ± 1:00 h:min (range 4-11 h). Overall, results showed that 19% of athletes slept less than 7 h, and 50% slept less than 8h. For all athletes sleep onset latency was 21 ± 17 min. Mean global PSQI score was 5.0 ± 2.4, with 55% of athletes experiencing poor sleep quality (global PSQI score ≥ 5).

Self-reported ideal bedtime, wake time and sleep duration were all significantly different (*p*<0.001) to actual self-reported sleep times. Average ideal bedtime (hh:min), wake time (hh:min) and sleep duration (hh:mm) was 22:22 ± 00:44 (range 20:00 - 02:00), 07:48 ± 00:58 (range 05:00-11:00) and 09:26 ± 00:58 (range 07:00 - 12:30 min), respectively.

### Male and female sleep behaviors

Self-reported current bedtime and wake time were significantly different in males compared to females, with males reporting a later bedtime and wake time (*p*=0.03 for both bedtime and wake time) (Table 3). No differences between males and females were found in sleep onset latency, current sleep duration or sleep quality (Table 3). In addition, no sex differences were found in any of the self-reported ideal sleep variables.

**Table 3:** Current self-reported sleep behaviors.

Sex	Sleep onset latency (min)	Bedtime (hh:mm)	Wake time (hh:mm)	Sleep Duration (hh:mm)	Global PSQI score
Males	21 ± 17	23:01 ± 00:52*	07:29 ± 01:03*	07:35 ± 01:03	5.0 ± 2.4
Females	24 ± 16	22:44 ± 00:57	07:10 ± 01:13	07:31 ± 01.07	5.4 ± 2.6

Values are mean ± SD.

* Significantly different to females (p<0.05).

### Age

A weak negative correlation was found between age and sleep duration (r=-0.30, *p*<0.001 and bedtime (r=-0.34, *p*<0.001). In addition, a moderate negative correlation was found between age and wake time (r=-0.49, *p*<0.001). No relationships was found between global PSQI score and age. When athletes were separated by age groups, the youngest age cohort (18-24 year old) were found to have a significantly later bedtime and wakeup time, and slept for significantly more hours than all other age groups (Table 4.). No differences in global PSQI scores were found between the different age groups. Differences were found between age cohorts and ideal sleep duration (Table 4), with the youngest cohort reporting significantly longer ideal durations compared to all others.

**Table 4:** Current self-reported sleep behaviors.

Age (y)	Bedtime (hh:mm)	Wake time (hh:mm)	Sleep duration (hh:mm)	Ideal sleep duration (h:min)	Global PSQI score
18-24	23:17 ± 00:52^a^	08:04 ± 01:07^a^	07:50 ± 00:58^a^	09:40 ± 00:58^a^	4.9 ± 2.3^a^
25-34	22:38 ± 00:46^b^	06:53 ± 00:50^b^	07:22 ± 00:58^b^	09:20 ± 00:56^b^	5.2 ± 2.5^a^
35-44	22:34 ± 00:49^b^	06:26 ± 00:50^b^	07:03 ± 01:04^b^	08:49 ± 00:44^c^	5.2 ± 2.7^a^
45-54	22:33 ± 00:38^b^	06:23 ± 00:41^b^	07:04 ± 00:48^b^	08:58 ± 00:35^bc^	4.1 ± 2.6^a^

Values are mean±SD. Groups sharing the same letter are not significantly different (p<0.05).

### Sport type

**Figure 1:**
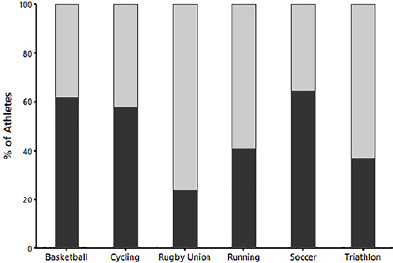
Percentage of athletes with a Global PSQI Score of ≤4 (dark grey) and ≥5 (light grey). A score ≥5 is indicative of poor sleep quality.

Data from the PSQI questionnaire revealed differences in self-reported bedtime, wake time and sleep duration between sports. Runners were found to sleep significantly less than basketballers, soccer players, rugby players, and cyclists (Table 5). No differences were found in sleep onset latency or in sleep quality between sports (Table 5). In, three out of the six sports, ≥50% of athletes had a global PSQI score of 5 or above, indicative of poor sleep quality. Rugby union players had the highest percentage (76%) of players with a global PSQI score above 5 (Figure 1).

**Table 5:** Current sleep behaviors by sport.

Sport	Sleep onset latency (min)	Bedtime (h:min)	Wakeup time (h:min)	Sleep duration (h:min)	Ideal sleep duration (h:min)	Global PSQI Score
Basketball	22 ± 20^a^	23:33 ± 01:02^a^	08:12 ± 00:54^a^	07:55 ± 00:56^a^	09:47 ± 01:02^a^	4.4 ± 2.6^a^
Cycling	16 ± 12^a^	22:37 ± 00:38^b^ ^c^	07:01 ± 0:58^bc^	07:39 ± 0:48^a^	09:24 ± 01:04^a^	4.6 ± 2.4^a^
Rugby union	23 ± 17^a^	22:44 ± 00:46^b^ ^c^	07:25 ± 1:02^b^	07:45 ± 0:47^a^	09:46 ± 01:00^a^	5.4 ± 1.9^a^
Running	22 ± 17^a^	22:51 ± 00:49^b^ ^c^	06:45 ± 1:06^c^	06:58 ± 1:05^b^	09:16 ± 00:56^a^	5.6 ± 3.2^a^
Soccer	22 ± 16^a^	23:31 ± 00:50^a^	08:26 ± 1:21^a^	07:50 ± 1:04^a^	09:13 ± 01:02^a^	4.7 ± 2.9^a^
Triathlon	21 ± 13^a^	22:27 ± 00:45^c^	06:40 ± 0:49^c^	07:16 ± 0:59^a^ ^b^	09:14 ± 00:52^a^	5.6 ± 2.6^a^

Values are mean±SD. Sports sharing same letter are not significantly different (p<0.05).

### Competitive level differences

On average elite and national athletes trained more times per week compared to competitive and recreational athletes (Table 2). Furthermore, elite athletes trained for longer during each training session compared to all other competitive levels (Table 2). No correlation was found between number of training sessions per week and sleep duration (*p*>0.05). A week positive correlation was found between sleep duration and duration of training (r=0.23, *p*<0.001). No correlation was observed between sleep latency and number of training sessions per week or duration of training.

Sleep duration grouped by competitive level can be found in Table 2. On average, self-reported sleep duration was significantly lower for recreational athletes compared to national and elite athletes. No differences in sleep onset latency or global PSQI score were found between athletes differing in competitive level (Table 2).

## DISCUSSION

This study quantified the self-reported current and ideal sleep duration and sleep quality in athletes varying in gender, age, sport type, and competitive level. The main findings were: 1) 19% of athletes reported to sleep less than 7h of sleep per night; 2) 50% of were found to have less than 8h sleep per night; 3) Runners and recreational athletes sleep less than other sport types and competitive levels, respectively; 4) Sleep quantity and quality did not differ between males and females, whilst the youngest cohort of athletes reported the longest sleep duration.

Our data taken from the PSQI questionnaire revealed that over half of the athletes had poor sleep quality (with a global PSQI score of ≥5). This finding is in agreement with previous studies who reported that 50% of professional ballet dancers and team sport athletes report a PSQI score of >5^20,21^. Sleep is considered a vital process for both health well-being and recovery^22^. Thus, inadequate sleep duration and quality may prevent athletes achieve peak performance and may dampen training adaptations^21^. In the present study, data were also collected on athletes’ current sleep duration, with 19% of athletes achieving less than the 7 h recommended by the National Sleep Foundation^3^. Furthermore, large disparities were found between self-reported current duration and ideal sleep duration. Specifically, longer ideal sleep duration was reported compared to current sleep duration. This was a result of athletes reporting an earlier ideal bedtime and a later wake time than their current behaviors. Although sleep requirements are likely specific to the individual, athletes will have a greater need for sleep (7-9 h/day)^23^. Failure to achieve these sleep durations may impact on the physiological and psychological recovery following training^23^.

### Competitive level differences

On average the sleep duration of the 313 athletes assessed in the current study was 7 h 24 min. This is longer than that reported by Lastella et al.^16^, who found average sleep duration to be ~6 h 48 min, from 124 elite athletes. Previously, no differences in sleep duration have been observed between elite athletes and non-athletes^24^. However, our data found recreational athletes to have significantly shorter sleep duration than national and elite level athletes. Speculatively, this group of athletes may have additional responsibilities outside of their sport (such as employment), which may dictate training times to early morning or late at night, thus reducing the available hours for sleep. In addition, the more elite competitor may also place a higher value on the importance of sleep and, therefore, prioritize it more than the recreational athlete. Nonetheless, further insights are required to identify barriers to sleep in this level of competitor.

In terms of sleep quality it has previously been reported that elite athletes have poorer sleep quality than non-athletes^24,25^. Although we reported recreational athletes to have shorter sleep durations we observed no differences in sleep quality between the four levels of competitive groups. A recent systematic review reported that elite athletes generally display signs of poor sleep quality due to longer sleep latencies and greater sleep fragmentation^7^. However, we found no differences in sleep latency between competitive levels. This was also reflected in the correlation data where we observed no association between number of training sessions and duration of training, despite elite and national athlete training more throughout the week. Although, in the present study, a crude method to measure sleep latency was used, and sleep fragmentation was not measured, therefore we were unable to detect if any differences in these variables exist between competitive levels.

### Sport differences

The present study compared sleep behaviors from athletes competing in six different sports. We observed that runners had a significantly shorter sleep duration than rugby union players, soccer players, basketballers, and cyclists. Numerous factors such as training or competition times, volume, intensity, period of the season (in-season vs. out of season), and load could account for these differences^20,26^. Nevertheless, the aforementioned reasons are purely speculative, as this information was not collected in the current study and therefore warrants further investigation. Our results support previous studies that report athletes competing in individual sports have a shorter sleep duration than team sport athletes^16^. Lastella et al.^16^ suggested that this was a consequence of earlier training sessions. This notion is supported by Sargent et al.^26^ who found reduced sleep in swimmers on training days due to the early start time of training^16^. The findings of the present study support this theory as we observed that athletes from individual sports (e.g., cycling, running, and triathlon) had earlier mean wake up times compared to those from team sports (soccer and basketball).

In terms of sleep quality, no differences were observed between sports. However, it is should be highlighted that in three out of the six sports used in this analysis, more than 50% of athletes had a global PSQI score indicative of poor sleep quality (≥5). As such, these data emphasize the need to assess the sleep quality of athletes on an individual basis and across all sports.

### Sex and age differences

In a recent study, a single night sleep was assessed using polysomnography in a cohort of 146 elite athletes. In this study, females were found to have better sleep quality than their male counterparts, with male athletes more likely to snore and have insomnia^27^. In the present study, female athletes had a significantly earlier bedtime and later wake time compared to males. However, no differences in self-reported sleep duration or sleep quality were observed. This discrepancy in results may indicate that females may be more attune to their sleep habits than males, who could speculatively underestimate their sleep. In support of this, Leeder et al.^25^ found time awake (the actual time spent awake determined from sleep start to sleep end) to be significantly greater in males, compared to females. In addition, lower sleep efficiency was found in males (~2.4% lower) compared to females^25^. However, the sex differences reported by Leeder et al.^25^ were only found when data from athletes and non-athletes were combined.

In terms of age differences, it was observed that athletes in the young cohort (18-24 y) had significantly longer sleep than all other ages. In addition, we found a weak negative correlation between age and sleep duration. Despite differences in sleep duration, we observed no differences in sleep quality between the different age groups. This is in contrast to an epidemiological study that also used the PSQI to assess sleep quality in 2,406 healthy adults. In this study, sleep quality decreased between 18 and 98 years of age^28^. It should be noted that the study by Gadie et al.^28^ was not conducted on athletes *per se*. In fact, exercise may help improve sleep quality in middle-aged and older adults^29^.

### Limitations

It is important to acknowledge the data in the present study were collected under different conditions for each athlete. For instance, the athletes’ training phase, schedule or any upcoming competitions have been unaccounted. Consequently, training load, intensity and frequency may have differed between athletes, all of which could impact sleep^26^. It should also be noted that at the time of data collected (2012-2014) no athlete specific sleep questionnaire was available. More recently, the Athlete Sleep Screening Questionnaire (ASSQ) and the Athlete Sleep Behavior Questionnaire (ASBQ) have been developed and validated^30,31,32^. Use of athlete specific sleep questionnaires are more sensitive to the unique challenges facing athletes and should be adopted for future research^30^.

In addition, the data was recorded via self-reported measures, which means that the results are subjective, and liable to bias. Thus, future research may use sleep activity monitors alongside sleep diaries, in order to offer quantifiable data and to provide more information on sleep efficacy, latency, and fragmentation. However, it is also important to note the limitations when using commonly available sleep ‘wearables’. Previous studies comparing actigraphy (from wearable sleep monitors) to the gold-standard method (polysomnography) have shown that wearable monitors have high sensitivity (the ability to correctly identify sleep) but low specificity (the ability to correctly identify wake^33,34,35^. Therefore, interpreting information from sleep tracking monitors should be made with caution. Finally, our data does not establish the implications of reduced sleep duration and/or quality on athlete training, competition and general well-being. Saner et al.^36^ reported lower muscle protein synthesis following 5-nights of sleep restriction (4h of sleep/night) but this was offset when a bout of high-intensity exercise was performed on the days when sleep was restricted^36^. Although these findings provide direct evidence on the interaction between sleep, protein synthesis and exercise, further research is needed to investigate the long-term effects of this relationship and the subsequent effect on exercise performance.

In conclusion, recreational athletes and athletes who participate in running sleep less than other competitive levels and sport types. In addition, 19% of all athletes self-reported a shorter sleep duration, than that recommended for healthy adults. Further research remains necessary to understand the barriers to better sleep in those athletes most at risk of not meeting guidelines, as well as the impact of improving sleep on athletic performance.
